# Structural disorder promotes assembly of protein complexes

**DOI:** 10.1186/1472-6807-7-65

**Published:** 2007-10-08

**Authors:** Hedi Hegyi, Eva Schad, Peter Tompa

**Affiliations:** 1 Institute of Enzymology, Biological Research Center, Hungarian Academy of Sciences, Budapest, Hungary

## Abstract

**Background:**

The idea that the assembly of protein complexes is linked with protein disorder has been inferred from a few large complexes, such as the viral capsid or bacterial flagellar system, only. The relationship, which suggests that larger complexes have more disorder, has never been systematically tested. The recent high-throughput analyses of protein-protein interactions and protein complexes in the cell generated data that enable to address this issue by bioinformatic means.

**Results:**

In this work we predicted structural disorder for both *E. coli *and *S. cerevisiae*, and correlated it with the size of complexes. Using IUPred to predict the disorder for each complex, we found a statistically significant correlation between disorder and the number of proteins assembled into complexes. The distribution of disorder has a median value of 10% in yeast for complexes of 2–4 components (6% in *E. coli*), but 18% for complexes in the size range of 11–100 proteins (12% in *E. coli*). The level of disorder as assessed for regions longer than 30 consecutive disordered residues shows an even stronger division between small and large complexes (median values about 4% for complexes of 2–4 components, but 12% for complexes of 11–100 components in yeast). The predicted correlation is also supported by experimental evidence, by observing the structural disorder in protein components of complexes that can be found in the Protein Data Bank (median values 1. 5% for complexes of 2–4 components, and 9.6% for complexes of 11–100 components in yeast). Further analysis shows that this correlation is not directly linked with the increased disorder in hub proteins, but reflects a genuine systemic property of the proteins that make up the complexes.

**Conclusion:**

Overall, it is suggested and discussed that the assembly of protein-protein complexes is enabled and probably promoted by protein disorder.

## Background

Intrinsically unstructured/disordered proteins or protein domains (IUPs) lack a well-defined structure, yet they carry out important functions [1-4]. IUPs often function by molecular recognition, when they bind partner molecules and undergo binding-induced folding transitions [5,6]. In these, the presence of protein disorder is thought to confer many functional advantages, such as the increased speed of interaction, specificity without excessive binding strength, and the adaptability to different partners, i.e. binding promiscuity or moonlighting [7]. These advantages may explain the recent observation that hub proteins, i.e. proteins involved in multiple interactions, tend to have a higher level of structural disorder than other proteins in the interactome [8-11], although the difference is small, and was not observed in one study [12].

Extending beyond these advantages is the suggestion that due to their open and exposed structure, IUPs might be able to simultaneously bind multiple partners [13], which enables the assembly of large complexes. Whereas disorder in such "assembler" functions [3,14] apparently has a significant advantage, its validity relies on a few isolated observations only. The high level and/or observed mechanistic role of disorder in the assembly process of the bacterial flagellum and viral capsid [15], the cytoskeleton, ribosome and clathrin coat [16,17], or some scaffolding proteins, such as BRCA1 and Ste5 [18,19], serve as focal points for the suggestion that structural disorder enables the assembly of large complexes. Whereas physical logic for such an assembly process implies large-scale structural rearrangements enabled by excessive flexibility, this inference has never been systematically tested. Recent high-throughput TAP-tag/MS studies of the full complement of protein-protein interactions (the interactome) of *E. coli *and *S. cerevisiae *[20-22] enabled us to probe into the general validity of the role of protein disorder in complexes and the assembly process.

Since the assembly of larger complexes may be conceived to process from smaller complexes at the expense of the burial of an increasing surface with increasing complex size, the foregoing considerations suggest that protein disorder should increase with increasing numbers of complexed proteins. We checked this inference by protein disorder prediction and also by looking for disorder in protein components of complexes in PDB. By applying IUPred [23,24], we found a statistically significant correlation between disorder and the number of proteins assembled into complexes for both *E. coli *and *S. cerevisiae*. The predicted correlation is also seen for a limited set of proteins for which experimental evidence of disorder can be found in the PDB. Our observations provide comprehensive evidence that the fraction of protein disorder increases with increasing complex size, which corroborates and extends previous suggestions that protein disorder is directly advantageous in protein-protein interactions [3,6,14].

## Results

### The population of the different size groups in *E. coli *and yeast complexes

First, we grouped the complexes in each dataset according to size. Initially, we formed 5 groups, with complexes containing 2–4, 5–10, 11–20, 21–30 and 31–100 proteins, respectively, and also added a group of singular proteins. In most cases the latter 3 groups of complexes were collapsed into one as most statistical tests (see below) did not show significantly different distributions for them. The singular proteins for yeast were exclusively derived from data by Gavin and coworkers [21] for which complex-forming was experimentally checked but none was found. For *E. coli*, we selected those Swiss-Prot proteins, for which neither in IntAct nor in Swiss-Prot was there any information about complex-forming, i.e. they did not appear in IntAct as part of a complex and there was no mentioning of the word "complex" in the annotation of these *E. coli *proteins in SwissProt. The population of each group in the different datasets for different organisms is shown in Tables 1 and 2, with respect to the number of complexes (Table 1) and the number of individual proteins (Table 2) for each group. The largest complex in yeast was found to consist of 95 proteins. In the *E. coli *datasets the largest complex had 64 proteins. No other organisms had sufficient numbers of complexes derived in a consistent fashion amenable to our analysis in the IntAct database or other sources. It should be noted that in the yeast data set most proteins (about 2/3rd of the total of 1490 proteins) occurred in more than one complex; and even in the *E. coli *data sets this was true of 35–50% of the total of complexed proteins.

**Table 1:** Number of complexes in the different data sets

Complex size	Yeast (Gavin06)	E. coli (Arifuzzaman06)	E. coli (Butland05)	E. coli (Gully06)
2 – 4 proteins	162	1017	257	165
5 – 10 proteins	136	747	89	115
11 – 20 proteins	98	172	46	74
21 – 30 proteins	146	14	22	38
31 – 100 proteins	60	2	10	24

For yeast, those proteins were deemed singular for which no binding partners have been found in each study (they were not cross-checked against each other). For *E. coli*, those proteins in Swiss-Prot were deemed singular (altogether 1922 proteins), for which neither in IntAct nor in the Swiss-Prot annotation lines did we find any evidence of complex-forming.

**Table 2:** Number of singular proteins and proteins in complexes of different sizes in the different data sets

Complex size	Yeast (Gavin06)	E. coli (Arifuzzaman06)	E. coli (Butland05)	E. coli (Gully06)
singular proteins	241			
2 – 4 proteins	346	1446	367	322
5 – 10 proteins	650	1573	226	441
11 – 20 proteins	789	827	186	382
21 – 30 proteins	647	187	194	325
31 – 100 proteins	747	57	128	245

For yeast, those proteins were deemed singular for which no binding partners have been found in each study (they were not cross-checked against each other). For *E. coli*, those proteins in Swiss-Prot were deemed singular (altogether 1922 proteins), for which neither in IntAct nor in the Swiss-Prot annotation lines did we find any evidence of complex-forming.

### Distribution of the predicted disorder of the complexes of different sizes

**Figure 1:**
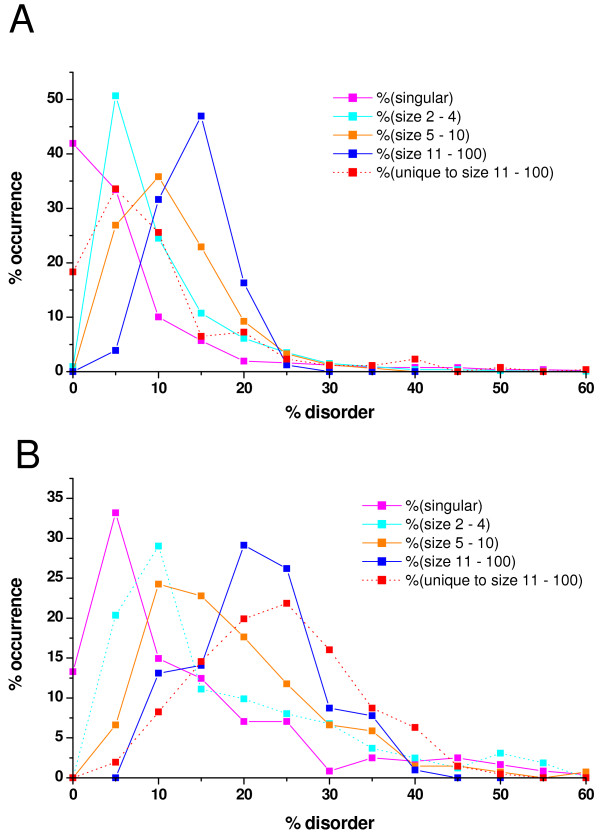
**Distribution of the complex-averaged disorder for complexes of different sizes**. The distributions are grouped by the sizes of complexes and singular proteins for which no evidence of complex-forming has been found. Magenta – singular proteins, cyan – complexes size 2–4, orange – complexes size 5–10, blue – complexes size 11–100, red: unique to complexes of size 11–100. The average disorder for each complex has been calculated by predicting the individual protein components and averaging them for each complex individually. **A**) *E. coli *complexes, taken from the IntAct database (significant differences among the different distributions with chi-square tests, p-value < 0.02). **B**) Yeast complexes (significantly different distributions, p-value < 0.01).

In the first approach we predicted the intrinsic disorder for each protein in all the complexes with the IUPred server, by counting all the disordered amino acids in each protein, then dividing it by the length of the protein in question. After calculating this relative disorder for each protein, we simply determined the disorder of each complex by averaging the relative disorder of the proteins contained in each complex. The distributions of the average disorder for complexes of various sizes for *E. coli *and yeast are shown in Figure 1. The distributions of complexes of various sizes are clearly different from one another and also from the uncomplexed group of proteins. As expected, there is a clear tendency for larger complexes to "peak" at greater relative disorder values. A chi-square test was performed to see if the differences in the distributions of the complexes of different sizes and also the group of "singulars" are significant. The results (not shown) indicated that from the perspective of disorder there are essentially 4 different groups in both species: singulars, complexes of size 2 to 4, 5 to 10 and complexes consisting of more than 10 proteins. The chi-square tests showed that the distributions of the four groups differed significantly in both species with a p-value < 0.001. As shown in Figs. 1A and 1B we also formed separate groups of those proteins that are unique to the large complexes in *E. coli *and yeast, respectively, (i.e. they occur exclusively in the large complexes consisting of more than 10 proteins) and calculated the average disorder of each large complex using only these unique proteins. It is clear that in yeast this subset is even more disordered than the average calculated from all the components of these complexes – e.g. the median value for the average percentage disorder of this unique complex subset increases from an average of 18 to more than 21 (data not shown). Interestingly, we did not find such an increase in the disorder of those proteins that were unique to the large complexes in *E. coli *(Fig 1A). While the absolute number of such proteins is rather small (Additional File 1A) compared to the other groups and also to yeast (additional file 1B), this fact in itself would not explain the difference between the two organisms. However, it might be explained by an evolutionary pressure on yeast to evolve such proteins over time (simultaneously with the appearance of larger complexes) whereas this did not seem to be the case for *E. coli*. The greater average disorder of larger complexes in the latter could probably be attributed to the larger relative weight of the more disordered pre-existing proteins in such complexes, but not to the incorporation of novel proteins of high level of disorder. This scenario of innovation in creating large complexes in yeast is entirely consistent with the observed sharp increase in the overall level of disorder upon going from prokaryotes to eukaryotes [17,25,26].

**Figure 2:**
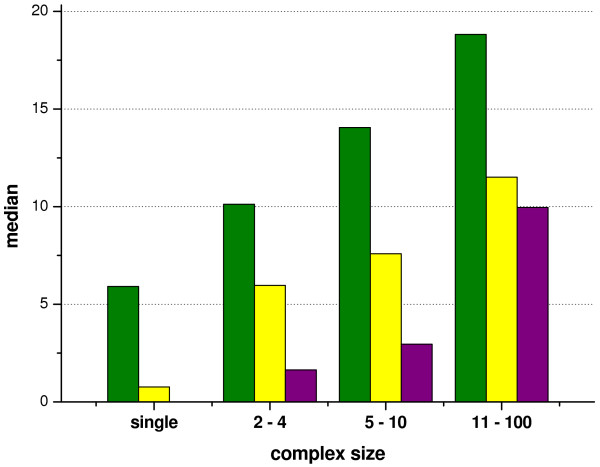
**Median values for predicted and observed disorder of complexes**. Median values were calculated for distributions of IUPred-predicted disorder of complexes of various numbers of components (Figure 1). Green – yeast predicted, yellow – *E. coli *predicted, purple – *E. coli *observed. For *E. coli*, we also recorded the complex average and median values for the different size groups derived from the observed values of matching PDB structures (with at least 90% sequence identity between the complex components and the PDB-s and almost full coverage, i.e. the length difference between the protein components and the PDB-s was less than 50 amino acids).

The median values for complexes of different sizes in both *E. coli *and yeast are shown in Fig. 2. The values show a clear distinction among the different groups in both species, with an increase in the median value with increasing complex size. (Throughout the paper we used the median rather than mean values as the median is less sensitive to outliers and consequently discriminated more among the different groups than the mean values.)

### Disorder calculated from segments of more than 30 residues

**Figure 3:**
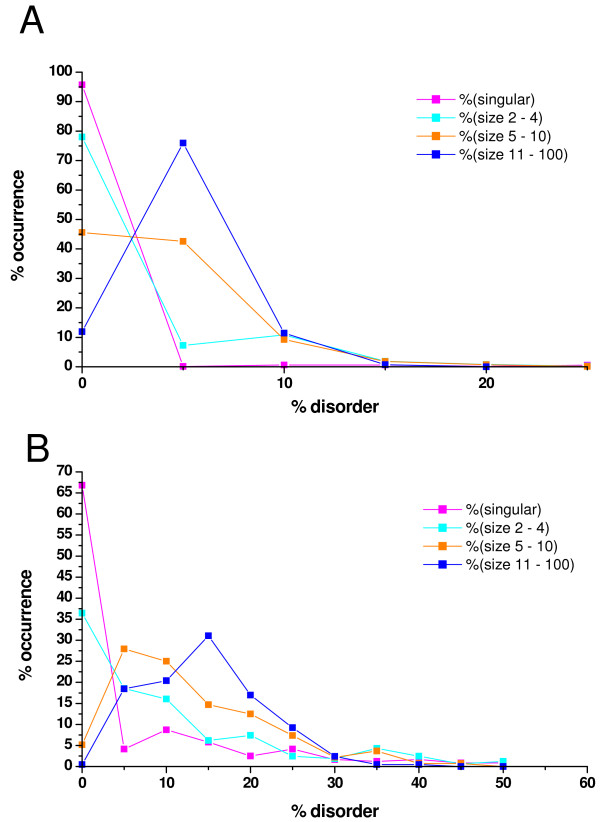
**Distributions of complex-averaged disorder of long disordered regions for complexes of different sizes**. The distributions are grouped by the sizes of complexes and singular proteins for which no evidence of complex-forming has been found. Magenta - singular proteins, cyan - complexes size 2-4, orange - complexes size 5-10, blue - complexes size 11-100. The average disorder for each complex has been calculated by predicting the individual protein components, and averaging them for each complex individually by considering only residues which fall into segments longer than 30 consecutive disordered residues. Complex averages were calculated and their % distributions are presented here. Medians are indicated in parentheses. **A**) E. coli complexes (significantly different distributions, p-value<0.001). **B**) Yeast complexes (significantly different distributions, p-value<0.001).

Regions of disorder of various lengths are conceptually distinguished in the literature, as the structural state of short disordered regions is more context-dependent, whereas that of long disordered regions is more context-independent. Whereas both kinds of regions are of clear functional significance, they may be involved in different kinds of functions [25,27-29]. Although not supported by comprehensive experimental evidence or theoretical considerations, the two classes are usually distinguished by a threshold length of about 30 amino acids, as also manifested in creating the predictor PONDR VSL2, which is composed of two separate predictors for short- and long disordered regions, based on this threshold value [28]. Because the assembly of large complexes is expected to depend more on such continuous sequences of disorder than on the average disorder of proteins, it is reasonable to ask if the presence of such regions correlates with the sizes of complexes. To this end, we determined the disorder of the complexes by taking into account only those stretches of amino acids that were predicted as disordered for at least 30 amino acids in an uninterrupted fashion. We got results similar to the previously described calculations (Figure 3) but the relative differences among the different groups are even more pronounced. For example, the median value more than triples upon going from the smallest (3.7% for complexes of 2–4 proteins) to the largest complexes (12.0% for complexes of 11–100 proteins) in the case of yeast.

### Distribution of the disorder of the protein components in the different complex size groups

**Figure 4:**
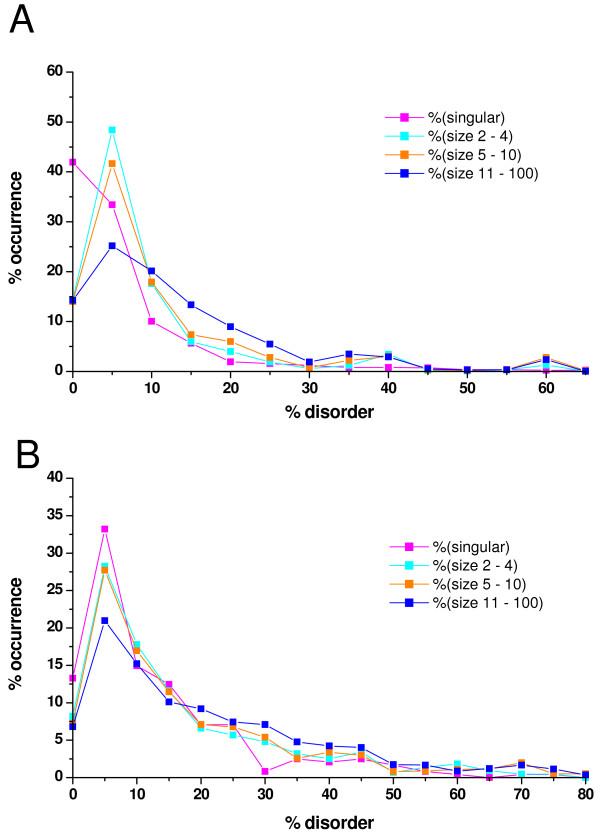
**Distribution of the relative disorder of the proteins in complexes of various sizes**. Individual proteins were taken from the complexes, their levels of disorder were predicted by the IUPred server, and are shown grouped according to the size of the complexes they were found in. Median values are indicated in parentheses. Magenta - singular proteins, cyan - complexes size 2-4, orange - complexes size 5-10, blue - complexes size 11-100. **A**) E. coli complexes (significantly different distributions, p-value<0.001, with the exception of difference between complexes of 2-4 and 5-10 proteins). **B**) Yeast complexes (significantly different distributions, p-value<0.001).

To see if the differences in the disorder of complexes of various sizes also show as differences in the disorder of the underlying proteins, we determined the distribution of the relative disorder of the proteins themselves in the different categories, using again the relative percentage disorder values predicted by IUPred. The results in Figure 4 (for both *E. coli *and yeast) show that while the distributions are similar, a chi-square test still can distinguish between most of them, especially if the proteins are taken into account as many times as they appear in the complexes of the targeted size range (as the same protein can appear several times in different complexes). Only the singular yeast proteins and those in small (size 2–4) complexes are not significantly different (p-value = 0.15), every other pair shows a statistically significant value (p-value = 0.02), in a sense that larger complexes use a greater proportion of more disordered proteins than smaller complexes. However, as shown in Figure 4, in all categories the most frequently occurring proteins were those with small (less than 10%) relative disorder.

### Distribution of the observed disorder of *E. coli *complexes derived from the PDB homologues of the protein components

The results presented thus far rely on disorder prediction by IUPred, which, at a false positive rate of 5% predicts disordered residues at a true positive rate of 76% [24]. To support these findings by actual data on disorder, we also compared the experimentally observed disorder of proteins in complexes of various sizes. To this end, we selected *E. coli *proteins in complexes that appear in PDB, using Blastp (yeast homologues were not numerous enough for a thorough statistical analysis). We used only those protein matches in PDB that had at least 90% sequence identity with a complex component. We considered almost full matches only where the lengths of the query proteins and that of the best match in PDB did not differ by more than 50 amino acids.

We used both single-chain and multi-chain PDB matches. Although in the latter set there might be significant disorder-to-order transition known to occur when a protein binds to its partner(s), still there is a linear relationship between the observed disorder (calculated as the average disorder of the PDB homologues of the complex components for each complex and the predicted one (additional file 2), which shows the relevance of disorder thus extracted from PDB.

**Figure 5:**
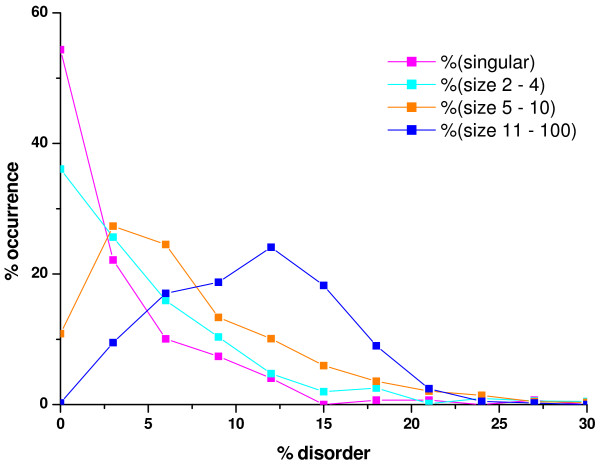
**Distribution of the experimentally observed average disorder of proteins in complexes of various sizes**. Averages of disorder were determined for those *E. coli *complexes, in which at least one component had an at least 90% sequence identity with a PDB chain whose length differed at most by 50 amino acids. Magenta – singular proteins, cyan – complexes size 2–4, orange – complexes size 5–10, blue – complexes size 11–100. The average disorder of the complexes was calculated by averaging the observed disorder of the matching PDB-s only.

The distributions of the observed disorder for the different size groups in *E. coli *are shown in Fig. 5. Although there is a smaller difference between smaller complexes (size 2–4) and singular proteins (with p-value = 0.033, according to a chi-square test) than derived from the predicted values, the smaller (size 2–4), the medium-sized (5–10) and larger (more than 10 components) complexes differ from one another significantly (with p-value < 0.0001). It should be stressed, that since we have taken from the PDB also proteins that are complexed, their observed disorder represents a lower limit of their actual level of disorder. This lends strong credit to our conclusions.

### Comparing complexes and hub proteins

The greater disorder of hub proteins (defined as such if they interact with more than 10 proteins in pair-wise interactions) observed by several groups independently [8-11]) raises the question of a relationship between hubs and complexes. The question is justified as there is a positive correlation between hub proteins and those that appear in a large number of complexes (additional file 3).

**Figure 6:**
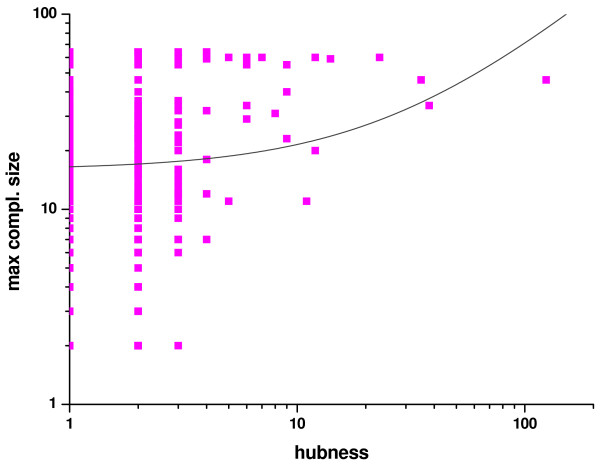
**The maximum complex size each *E. coli *protein occurs in, as a function of its "hubness"**. Only those *E. coli *proteins are presented here that appear in both pairwise interactions and complexes in the IntAct database (852 proteins altogether). For each protein the size of the largest complex it appears in is presented as the function of the number of interacting partners in pairwise interactions. Data are presented on a log-log scale. The fitted curve represents a linear relationship but a negligible one, apparent from the very small value of R^2^, 0.02.

On the other hand, there was no significant relationship between the size of the complex a protein appears in and its "hubness" (i.e. the number of its partners in pair-wise experiments) for either *E. coli *or yeast (shown only for *E. coli*, Fig. 6). This shows that the larger complexes-greater disorder relationship and the more pair-wise interactions-greater disorder [8-11] are most certainly two distinct phenomena, not directly related to each other.

## Discussion

It is traditionally held that a major advantage of protein disorder is that it facilitates protein-protein interactions, which may explain the increased level of disorder in hub proteins [8-11], and in some large protein complexes [15-17]. Since a large body of data on the identity of complexes of various sizes in *E. coli *and yeast has been generated in recent high-throughput TAP-tag/MS studies [20-22], we have been able to test if, as expected, larger complexes have more average disorder than smaller ones.

Our findings validate the expected correlation. For both *E. coli *and yeast, there is a statistically significant increase in average predicted disorder with the number of components of complexes. This increase is even more pronounced if only long (more than 30 consecutive residues) disordered regions are taken into consideration, which suggest that these regions are particularly relevant in protein-protein interactions, and in particular in the assembly of large complexes. The major source of the observed correlation is that larger complexes are assembled from proteins that tend to be more disordered, underlined by the observation that proteins unique to large complexes in yeast show the highest level of disorder. The observed correlation is also corroborated by experimentally observed disorder of individual proteins, selected from the PDB, even though traditional structure-solution and deposition in PDB is biased against long disordered regions [30].

The observed correlation points to important functional implications of protein disorder in the organisation and evolution of the interactome, and it also raises interesting experimental ideas and provide important functional insight. These points will be discussed next. First, our report relies mostly on the prediction of disorder, and requires further corroboration by experimental data. Undoubtedly, as the interactome research advances and more complexes are identified, these studies may be further refined. Also, because PDB is highly biased against disorder, alternative data sources providing data on the disorder of individual proteins will give a boost to these ideas. Such data are deposited into the DisProt database [31], which is expected to grow rapidly as protein disorder is gaining general recognition. Second, the observed correlation between disorder and complex size provides a mechanistic insight into the roles disorder plays in the assembly of complexes. One possibility is that disordered regions are involved in the binding process directly, as suggested already by the fact that local disorder serves recognition functions in the form of molecular recognition features (MoRFs [32]) or short linear motifs (SLMs [29]). Alternatively, disorder might provide flexible linkers of well-folded interaction domains, which might enable their productive interactions. These alternatives might be experimentally tested as the structures of more and more large complexes are solved. By the same token, as longer disordered segments appear to correlate better with complex size, this feature might also be experimentally tested.

The third ramification of our observations is in the evolution of protein complexes. If we consider the formation of large complexes in evolutionary terms, they must have come about by the addition of new components to smaller pre-existing complexes. The predicted increase in disorder with complex size is only compatible with this model if we assume that the newer proteins attached to complexes are more disordered, thus increasing the average disorder as observed. Whereas this inference will also become testable as actual structures of large complexes become available, it is already in agreement with the observed advance of protein disorder with evolution, i.e. the increase of observed disorder with evolutionary complexity of organisms, which suggests that newer proteins tend to be more disordered [17,25,26].

A further key point to address is the relationship of increasing disorder with complex size and an increased level of disorder in hub proteins [8-11]. Hubs in general organize the interactome, and "party" hubs [33] are involved in binding several partners at the same time, i.e. scaffolding (large) complexes. Our analysis, however, shows that the number of interacting partners is in no correlation with the number of components of complexes the protein is in, which suggests that increasing disorder with complex size is independent of the presence of hubs, and is probably a genuine property of the entire complex. This finding is in line with several previous observations. First, a significant fraction of hubs termed "date" hubs [33] are involved in binding multiple partners on distinct occasions, i.e. they increase the level of disorder of small complexes, but not that of large ones. The other point is that the level of disorder in hub(s) is not much larger than that in non-hubs ([8-10], and cf. a counterexample, [12]), and the presence of one such organizing protein does not necessarily increase the average level of the disorder of a complex. A final point is that it was suggested that often hubs are not disordered, but instead interact with disordered partners [34]. Incorporation of such a hub in a complex would decrease average disorder, and thus an increased level of disorder of the complex would rather reflect the disorder of interacting partners. In all, it appears our observation on complexes is a novel manifestation of the role of disorder in protein-protein interactions.

In conclusion, our studies provide evidence for the intimate link between protein disorder and the assembly of complexes. Larger complexes appear to have more average disorder and more of long segments of disorder, which are in perfect agreement with prior suggestions that a major evolutionary and functional asset of protein disorder is its involvement in protein-protein interactions [6,13]. Whereas our observations provide evidence for these previous suggestions, they also raise new and testable hypotheses, which will lead to novel experiments in future studies on protein disorder and protein-protein interactions.

## Methods

### *E. coli *and yeast datasets

We analyzed several datasets in the IntAct database [35] containing data about protein complexes in *E. coli *and *S. cerevisiae*, generated by TAP-tag/MS analysis. However, data obtained by yeast two-hybrid analysis have not been considered because complex size cannot be adequately inferred from pair-wise interaction studies. For *E. coli *we focused on three data sets, each containing experimental TAP-tag data on a large scale [22,36,37]. For yeast, we analyzed only one data set [21] present in IntAct, as this was the only data set that contained a reasonable number of large complexes determined from a single proteome-wide experiment. Unless otherwise noted, we refer to this experiment when we talk about yeast complexes throughout the paper.

### Prediction of disorder

We used the IUPred server [23] to predict the disorder of the *E. coli *and yeast proteins in the study. We determined the percentage disorder for each protein in the complexes by counting the number of disordered amino acids as predicted by IUPred, divided it by the length of the protein in question and multiplied the result with 100. We determined the average percentage disorder for each complex by averaging the percentage disorder of the component proteins of the complexes. These primary data can be found in additional files 4 and 5. For all these and the following steps we used in-house Perl scripts.

### Percentage distribution of percentage disorder

After determining the average percentage disorder for each complex we grouped them into different categories according to their size (i.e. the number of component proteins). For each category we determined the distribution of their disorder by counting the number of complexes in each disorder range (usually in increments of 5% of disorder) and normalizing each distribution curve in a way that the total number of values for each curve would add up to 100.

### Chi-square tests

To determine if the distributions of two different size ranges in a graph are significantly different we used the chi-square test as follows:

χ2=∑i=1n(Oi−Ei)2Ei
 MathType@MTEF@5@5@+=feaafiart1ev1aaatCvAUfKttLearuWrP9MDH5MBPbIqV92AaeXatLxBI9gBaebbnrfifHhDYfgasaacH8akY=wiFfYdH8Gipec8Eeeu0xXdbba9frFj0=OqFfea0dXdd9vqai=hGuQ8kuc9pgc9s8qqaq=dirpe0xb9q8qiLsFr0=vr0=vr0dc8meaabaqaciaacaGaaeqabaqabeGadaaakeaaiiGacqWFhpWydaahaaWcbeqaaiabikdaYaaakiabg2da9maaqahabaWaaSaaaeaacqGGOaakcqWGpbWtdaWgaaWcbaGaemyAaKgabeaakiabgkHiTiabdweafnaaBaaaleaacqWGPbqAaeqaaOGaeiykaKYaaWbaaSqabeaacqaIYaGmaaaakeaacqWGfbqrdaWgaaWcbaGaemyAaKgabeaaaaaabaGaemyAaKMaeyypa0JaeGymaedabaGaemOBa4ganiabggHiLdaaaa@4354@

where O_i _and E_i _are the observed and expected frequencies, respectively, for a series of data.

### PDB homologs of the complex components

We used Blastp [38] to compare the sequences of the *E. coli *and yeast proteins to those in PDB [39]. We took into account only those proteins in PDB that matched one of the protein components of the complexes or any of the non-complexed proteins in question with at least 90% sequence identity. If several proteins satisfied these criteria, only the best match was taken into consideration. We considered almost full matches only, where the lengths of the query proteins and that of the best match in PDB did not differ by more than 50 amino acids. This resulted in insufficient number of proteins for yeast, and thus we only carried out the subsequent analysis in the case of *E. coli *proteins. We assigned the disorder of each matching protein in the PDB by adding up the number of amino acids in the header of each PDB entry whose structure the authors could not determine, marked as "missing residues". However, we did not consider undetermined side chains as disordered.

### Selecting single proteins in yeast and *E. coli*

The list of single proteins in yeast was kindly provided by AC Gavin, defined as such as those proteins that were found not to be complexed with any other in the TAP-tag experiments by [21]. They found 241 such proteins. For *E. coli *we selected those proteins in SwissProt that did not have any evidence to be part of a complex according to either the IntAct database or Swissprot. We identified 1922 such proteins of the 4933 *E. coli *proteins present in SwissProt.

## Authors' contributions

HH carried out the database studies and automated data analysis and wrote Perl scripts to handle the data and wrote the Methods and Results sections. ES did the original pilot studies for the project. PT conceived the project and wrote Introduction and Discussion. All authors read and approved the final manuscript.

## Supplementary Material

Additional File 1 Distribution of the actual numbers of complex-averaged disorder for different size categories **A**) for E. coli and **B**) yeast. The distributions are the same as in Fig 1A and 1B but instead of normalizing the data to 100%, the actual numbers are presented for each category. Color codes: Magenta – singular proteins, cyan – complexes size 2–4, orange – complexes size 5–10, blue – complexes size 11–100, red: unique to complexes of size 11–100. Click here for file

Additional File 2 The average component %IU determined with IUPred versus the average component disorder (%unstr) observed in matching PDB chains for E. coli complexes. Only those components are taken into account that have a matching PDB chain when calculating the average %IU or the average %unstructuredness. Only those complexes are presented here that had at least 11 components matching a PDB chain (regardless of how many components that complex had altogether). Click here for file

Additional File 3 The number of complexes vs. the number of interacting partners for E. coli proteins. The number of different complexes versus the number of interacting partners in pairwise interactions (i.e. the "hubness") for each E. coli protein it participates in. The high value of R^2^, 0.60, indicates a strong correlation between the two variables. Click here for file

Additional File 4 Calculated and observed disorder for each ***E. coli ***protein present in a complex and also in PDB. Click here for file

Additional File 5 Average predicted disorder for each **yeast **complex. Click here for file
