# Supplementary figures and images for: Structural disorder promotes assembly of protein complexes
Source: BMC Struct Biol. 2007 Oct 8;7:65. doi: 10.1186/1472-6807-7-65 (PMC2194777; doi:10.1186/1472-6807-7-65)

A

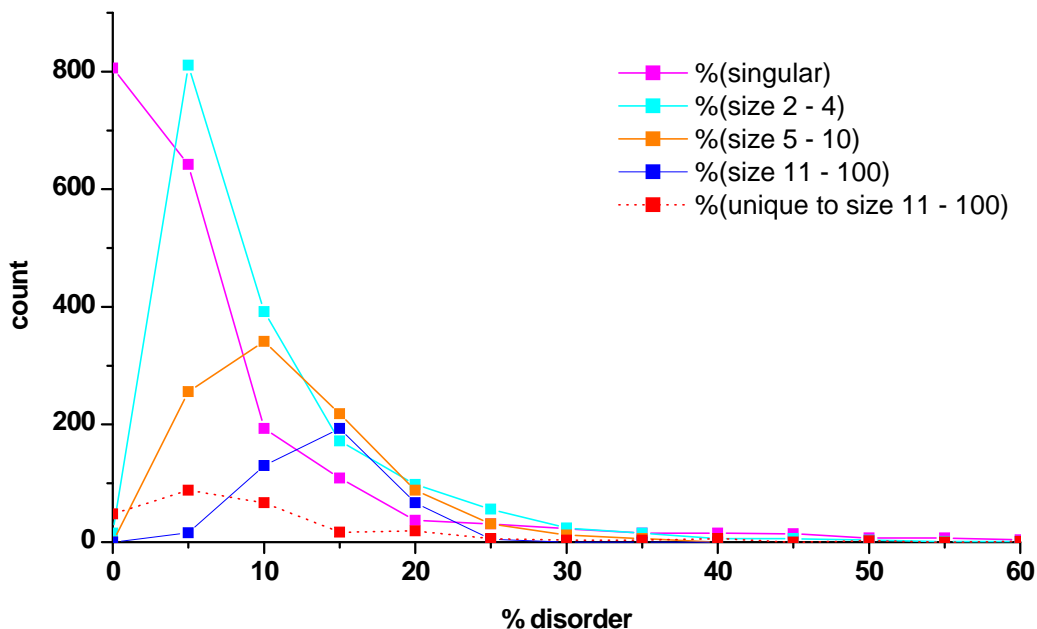

B

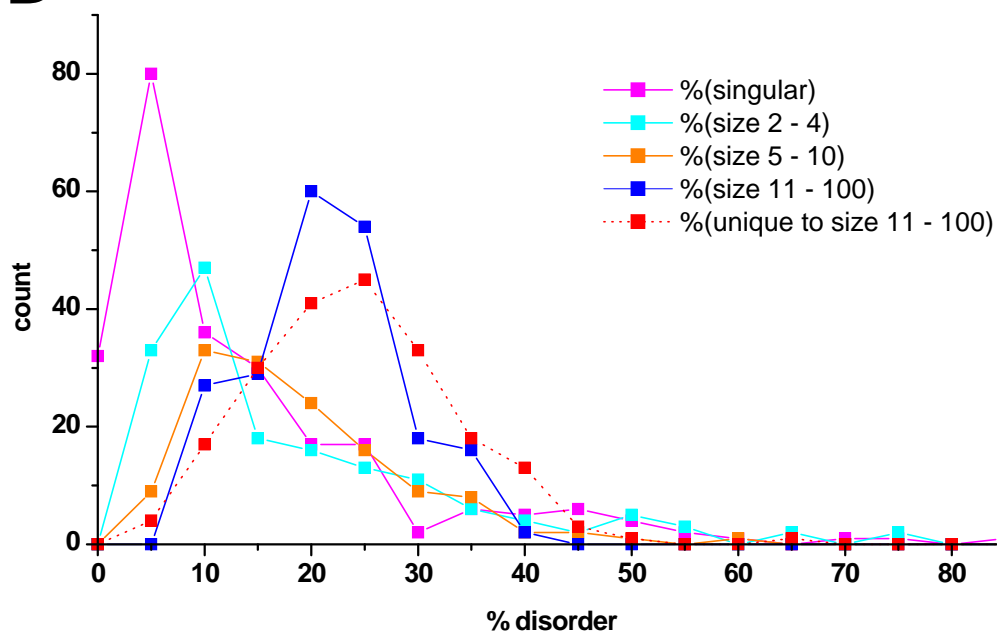

Supplement: Additional File 1 — Distribution of the actual numbers of complex-averaged disorder for different size categories A) for E. coli and B) yeast. The distributions are the same as in Fig 1A and 1B but instead of normalizing the data to 100%, the actual numbers are presented for each category. Color codes: Magenta – singular proteins, cyan – complexes size 2–4, orange – complexes size 5–10, blue – complexes size 11–100, red: unique to complexes of size 11–100. [file 1472-6807-7-65-S1.pdf]

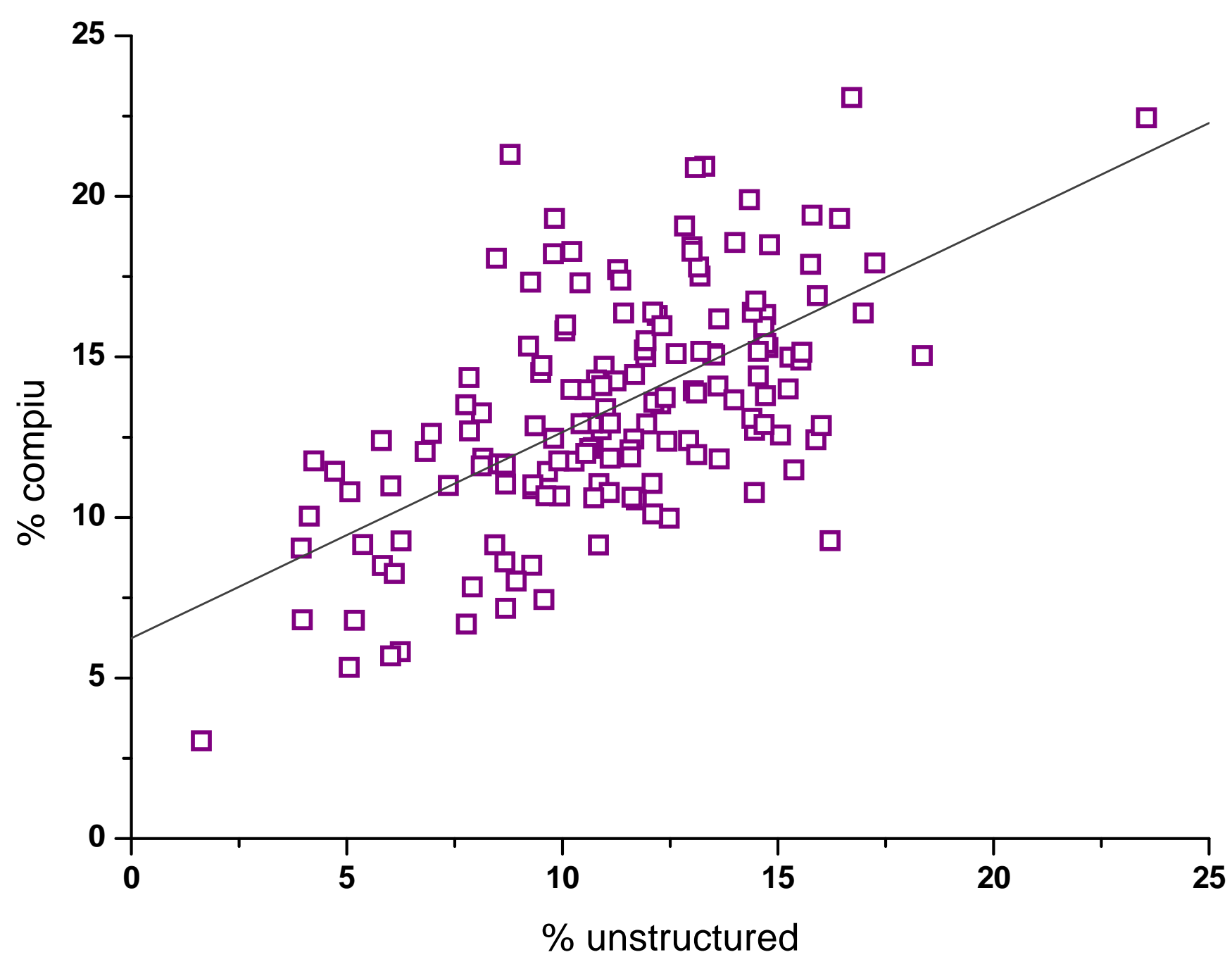

Supplement: Additional File 2 — The average component %IU determined with IUPred versus the average component disorder (%unstr) observed in matching PDB chains for E. coli complexes. Only those components are taken into account that have a matching PDB chain when calculating the average %IU or the average %unstructuredness. Only those complexes are presented here that had at least 11 components matching a PDB chain (regardless of how many components that complex had altogether). [file 1472-6807-7-65-S2.pdf]

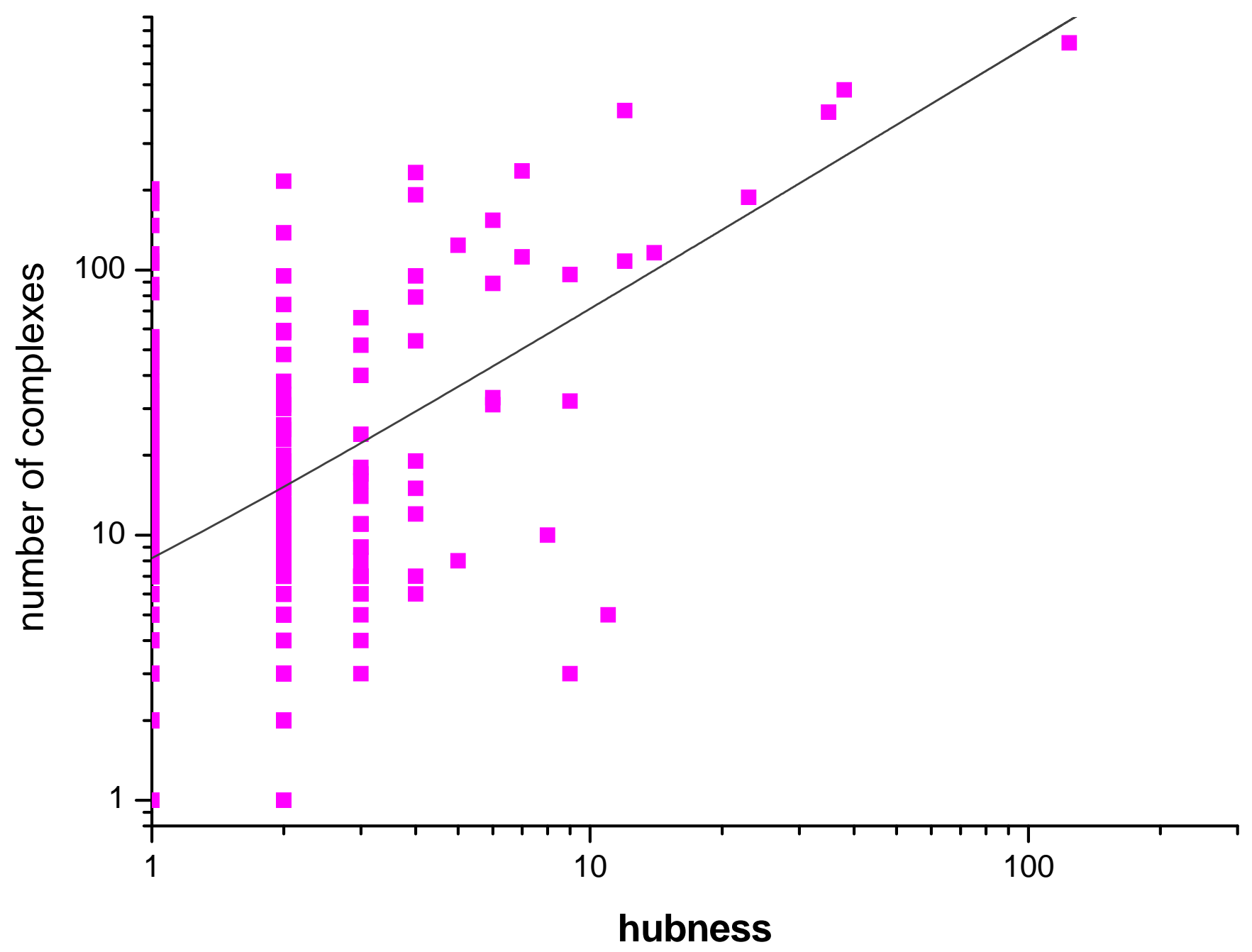

Supplement: Additional File 3 — The number of complexes vs. the number of interacting partners for E. coli proteins. The number of different complexes versus the number of interacting partners in pairwise interactions (i.e. the "hubness") for each E. coli protein it participates in. The high value of R2, 0.60, indicates a strong correlation between the two variables. [file 1472-6807-7-65-S3.pdf]
